# A tandem array of *CBF*/*DREB1* genes is located in a major freezing tolerance QTL region on *Medicago truncatula* chromosome 6

**DOI:** 10.1186/1471-2164-14-814

**Published:** 2013-11-21

**Authors:** Nadim Tayeh, Nasser Bahrman, Hélène Sellier, Aurélie Bluteau, Christelle Blassiau, Joëlle Fourment, Arnaud Bellec, Frédéric Debellé, Isabelle Lejeune-Hénaut, Bruno Delbreil

**Affiliations:** Université Lille 1, UMR 1281 Stress Abiotiques et Différenciation des Végétaux cultivés (SADV), Bâtiment SN2, F-59655 Villeneuve d’Ascq Cedex, France; INRA, UMR 1281 Stress Abiotiques et Différenciation des Végétaux cultivés (SADV), Estrées-Mons, BP 50136, F-80203 Péronne Cedex, France; INRA, Centre National de Ressources Génomiques Végétales (CNRGV), BP 52627, F-31326 Castanet-Tolosan Cedex, France; INRA/CNRS, UMR 441/2594, Laboratoire des Interactions Plantes-Microorganismes (LIPM), BP 52627, F-31326 Castanet-Tolosan Cedex, France

**Keywords:** Freezing tolerance, *Medicago truncatula*, Quantitative trait locus (QTL), Fine mapping, Candidate genes, *CBF*/*DREB1* genes, Tandem duplication, Sequence polymorphism

## Abstract

**Background:**

Freezing provokes severe yield losses to different fall-sown annual legumes. Understanding the molecular bases of freezing tolerance is of great interest for breeding programs. *Medicago truncatula* Gaertn. is an annual temperate forage legume that has been chosen as a model species for agronomically and economically important legume crops. The present study aimed to identify positional candidate genes for a major freezing tolerance quantitative trait locus that was previously mapped to *M. truncatula* chromosome 6 (Mt-FTQTL6) using the LR3 population derived from a cross between the freezing-tolerant accession F83005-5 and the freezing-sensitive accession DZA045-5.

**Results:**

The confidence interval of Mt-FTQTL6 was narrowed down to the region comprised between markers MTIC153 and NT6054 using recombinant F_7_ and F_8_ lines. A bacterial-artificial chromosome (BAC) clone contig map was constructed in an attempt to close the residual assembly gap existing therein. Twenty positional candidate genes including twelve C-repeat binding factor (CBF)/dehydration-responsive element binding factor 1 (DREB1) genes were identified from BAC-derived sequences and whole-genome shotgun sequences (WGS). *CBF*/*DREB1* genes are organized in a tandem array within an approximately 296-Kb region. Eleven *CBF*/*DREB1* genes were isolated and sequenced from F83005-5 and DZA045-5 which revealed high polymorphism among these accessions. Unique features characterizing *CBF*/*DREB1* genes from *M. truncatula*, such as alternative splicing and large tandem duplication, are elucidated for the first time.

**Conclusions:**

Overall, twenty genes were identified as potential candidates to explain Mt-FTQTL6 effect. Their future functional characterization will uncover the gene(s) involved in freezing tolerance difference observed between F83005-5 and DZA045-5. Knowledge transfer for breeding improvement of crop legumes is expected. Furthermore, *CBF*/*DREB1* related data will certainly have a large impact on research studies targeting this group of transcriptional activators in *M. truncatula* and other legume species.

**Electronic supplementary material:**

The online version of this article (doi:10.1186/1471-2164-14-814) contains supplementary material, which is available to authorized users.

## Background

Freezing is an adverse abiotic factor with severe negative impacts on plant health and productivity [1]. Most temperate plants can increase their freezing tolerance after exposure to low temperatures, a process called cold acclimation [1–3]. *CBF*/*DREB1* genes play a key role in the regulation of the transcriptome during cold acclimation [4]. They belong to the AP2/EREBP family of transcription factors and were first isolated from *Arabidopsis thaliana* (L.) Heynh. [5–7]. Up to now, *CBF*/*DREB1* genes have been identified in numerous herbaceous and woody plant species [8–11] and different studies have reported their significant role in freezing tolerance [12–16].

Grain legumes are an important source of food and feed worldwide. With their seeds containing 20% to 30% protein, they largely contribute to the protein requirements of humans and animals [17]. Forage legumes are a valuable source of protein, fiber and energy for livestock as well [17]. Besides their nutritional importance, legumes are an exceptional component to sustainable agriculture. In fact, most legume species are able to establish nitrogen-fixing symbioses with rhizobial bacteria [17, 18] which reduces the need for fertilizer application in crop rotation systems. Nowadays, the susceptibility to low temperatures and freezing conditions still represents a major constraint to the cultivation of legumes in many agro-ecological zones. The identification of the genes responsible for the natural freezing tolerance variation can enhance the breeding progress and subsequently the release of novel freezing-tolerant cultivars.

*Medicago truncatula* is an annual legume of Mediterranean origin [19]. It is not only a valuable forage crop as in Southern Australia [20] but has been chosen as a model molecular-genetic system for legume biology [21]. A large array of genomic tools has been developed for the reference accession A17 [22] and a draft sequence of the euchromatic portion of its genome comprising approximately 94% of all genes has been released [23]. These resources can accelerate the identification of the molecular determinants of various traits in *M. truncatula* including freezing tolerance. Knowledge can be then transferred to different agronomically and economically important crops considering their phylogenetic closeness to *M. truncatula* and the remarkable synteny between their genomes and that of *M. truncatula*[24–28].

Studies on cold acclimation and freezing tolerance in *M. truncatula* are scarce [29–34]. Few biochemical changes have been reported in response to low non-freezing temperatures for this species [30, 31, 34]. At the molecular level, transcripts corresponding to *M. truncatula* cold acclimation-specific gene 15, a candidate *CBF*/*DREB1* target, have been found to accumulate 6 hours after exposure to low temperature [32]. Four *CBF*/*DREB1* genes, i.e. *MtCBF1*-*4*, have been reported to be rapidly induced in response to cold stress [32, 34, 35]. Furthermore, the overexpression of *MtCBF3* (also named *MtDREB1C*) has been shown to improve the freezing tolerance of transgenic *M. truncatula* accession Jemalong [14]. *MtCBF1* and *MtCBF4* are located on *M. truncatula* chromosomes 5 and 1, respectively, but no information on the physical localization of *MtCBF2*-*3* has yet been obtained. Apart from *MtCBF1*-*4*, no other *CBF*/*DREB1* genes have been described and characterized from *M. truncatula* even though expressed sequence tag (EST) and complementary DNA (cDNA) potentially representing transcripts from other putative *CBF*/*DREB1* genes are available [36, 37].

Recently, Avia et al. [38] have uncovered natural variation for freezing tolerance among 15 *M. truncatula* accessions after a cold acclimation period. The genetic bases for freezing tolerance variation between the contrasted accessions F83005-5 and DZA045-5 have been identified using a quantitative trait locus (QTL) mapping approach [38]. A major freezing tolerance QTL (Mt-FTQTL6) accounting for 40% of the phenotypic variation has been mapped to a region of *M. truncatula* chromosome 6 [38] coinciding with an assembly gap in the A17 euchromatic sequence [39]. The present study aimed to fine map Mt-FTQTL6 and to identify genes located in the corresponding genomic region.

## Results

### Freezing tolerance evaluation of recombinant lines validates Mt-FTQTL6 position and narrows down its confidence interval

**Figure 1:**
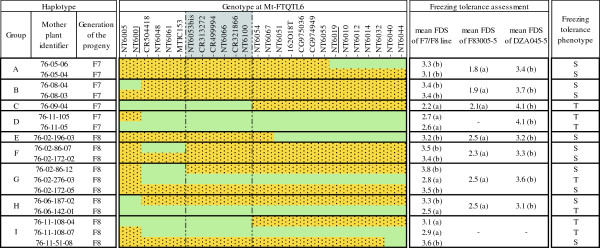
**Phenotypic evaluation of different haplotypes carrying or not recombination events within/next to the confidence interval of Mt-FTQTL6.** F_7_ and F_8_ seedlings were obtained from the self-pollination of the homozygous F_6_ and F_7_ lines whose code names are indicated in the ‘Mother plant identifier’ column. These lines originally derive from a cross between *M. truncatula* accessions F83005-5 [freezing-tolerant] and DZA045-5 [freezing-sensitive] (see ‘Development of plant material’ in Methods). The genotypes of F_7_/F_8_ lines at Mt-FTQTL6 are provided based on information from 26 markers that are shown according to their established genetic and/or physical order ([39]; this study). Green bars refer to chromosomal regions harboring alleles from F83005-5. Orange dotted bars represent regions with alleles from DZA045-5. Markers bordering the confidence interval of Mt-FTQTL6 according to [39] are underlined. These are 5.7 cM distant [39]. Haplotype groups A-I contain the recombinant and non-recombinant F_7_/F_8_ lines that were compared between each other. The mean freezing damage score (FDS) of each line is provided in the ‘Freezing tolerance assessment’ part of this figure. Pairwise comparisons of mean FDS were conducted between recombinant lines, respective controls (if any), F83005-5 and DZA045-5 (see ‘Evaluation of freezing tolerance’ in Methods). Letters (a) and (b) are used to distinguish significantly different FDS in each haplotype group. Due to the lack of individuals representing the parental accessions, the mean FDS of the progeny of recombinant line 76-11-108-04 in group I was only compared with those of the progenies of 76-11-108-07 and 76-11-51-08, considered as positive and negative controls respectively. Deduced phenotypes of F_7_/F_8_ lines are indicated to the right of the figure: ‘S’, sensitive to freezing; ‘T’, tolerant to freezing. Grey-highlighted markers in the ‘Genotype at Mt-FTQTL6’ part are those contained in Mt-FTQTL6’s confidence interval delimited according to genotype and phenotype data from screened lines.

**Figure 2:**
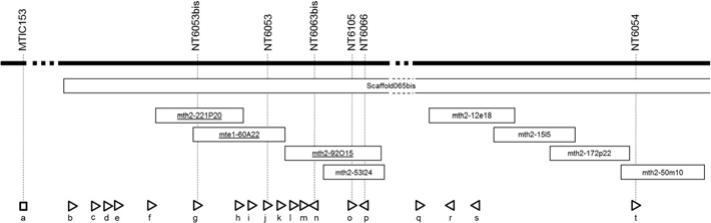
**Genetic and physical map positions of Mt-FTQTL6 candidate genes on** ***M*** **.** ***truncatula*** **chromosome 6.** Genetic markers shown at the top of the figure refer to gene-based markers bordering or located in the confidence interval of Mt-FTQTL6 (refer to Figure 1 and Additional file 2). BAC clones originating from the genomic region corresponding to Mt-FTQTL6 are presented according to their relative positions and with respect to genetic markers (see Additional file 5 for details on the BAC clone contig map). The identifiers of the BAC clones whose inserts were sequenced in this study are underlined. Scaffold065bis is a WGS scaffold from A17 that largely covers the confidence interval of Mt-FTQTL6. Arrowheads indicate the position and the transcriptional orientation of candidate genes. No information on the transcriptional orientation of *MtBAG*-*1* is available. a, *MtBAG*-*1*; b, *MtCBF14*; c, *MtCBF13*; d, *MtCBF3*; e, *MtCBF12*; f, *MtCBF11*; g, *MtCBF2*; h, *MtCBF10*; i, *MtCBF9*; j, *MtCBF8*; k, *MtCBF7*; l, *MtCBF6*; m, *MtCBF5*; n, *MtPERLD*; o, *MTR*_*050s0019*; p, *MTR*_*050s0020*; q, *MtZFWD*; r, *MTR*_*054s0001*; s, *MTR*_*054s0019*; t, *MTR*_*6g089580*.

Eighteen F_7_ or F_8_ lines were evaluated for freezing tolerance after a cold acclimation period (Figure 1). Based on markers used in this study (Additional file 1), screened lines correspond to 9 different haplotypes; 7 of which carry recombination events within or next to Mt-FTQTL6 confidence interval (Figure 1). Phenotypic data from F83005-5 and DZA045-5, included as check plants, were significantly different as expected. Comparisons of the phenotypes and marker genotypes validated Mt-FTQTL6 confidence interval previously located between markers NT6001 and NT6019 [39]. Freezing damage scores from the self-pollinated progenies of lines 76-08-04 and 76-06-187-02 showed that F83005-5 inserts upstream NT6001 have no phenotypic effect. The same conclusion was reached concerning F83005-5 inserts downstream NT6019 based on the progenies of line 76-05-06. Furthermore, Mt-FTQTL6 interval could be delineated to a smaller region of 0.4 cM between markers MTIC153 and NT6054 (Figure 1 and 2; Additional file 2). A critical recombination event in line 76-02-86, as seen in lines 76-02-86-07 and 76-02-86-12, suggested MTIC153 as the left marker for Mt-FTQTL6 confidence interval. The right marker, NT6054, was identified based on information from the progenies of lines 76-09-04 and 76-11-108-04.

### Full-BAC sequencing and WGS assembly provide sequences spanning the assembly gap coinciding with Mt-FTQTL6

Efforts have been made to close the assembly gap between markers NT6001 and NT6019. Six primary BAC clone contigs partly spanning Mt-FTQTL6 could thus be constructed (Additional files 3, 4, 5). Mt-FTQTL6 confidence interval delimited through fine mapping (see above) is partly covered by BAC clone contig IV (Additional file 5). In order to generate the candidate genomic sequence of Mt-FTQTL6, inserts from BAC clones mth2-92O15, mte1-60A22 and mth2-221P20, were first fully-sequenced (GenBank accession numbers [GB acc] KF006382-84). Sequence information from these 3 clones (Figure 2) extended BAC clone mth2-53l24 insert sequence (GB acc AC229695) with 217,951 bp assembled in 17 contigs (Additional file 6). With Illumina WGS sequences becoming available from A17 (F. Debellé, personal communication), BAC by BAC sequencing has not been kept up. Instead, marker and BAC-end sequences associated with Mt-FTQTL6 were used to search for scaffold sequences corresponding to the target region. A scaffold of more than 1 Mb long, namely scaffold065bis, was thus identified. It covers BAC clone contigs IV and V (Additional file 5) and extends mth2-221P20 insert sequence with approximately 155 Kb (Figure 2).

### Twenty non-transposon genes reside in Mt-FTQTL6 confidence interval

Twenty non-transposon genes with significant matches to GenBank’s non-redundant nucleotide (nr/nt), expressed sequence tag and protein databases, including those corresponding to markers MTIC153 and NT6054, could be identified in the candidate sequence for Mt-FTQTL6 (Figure 2; Table 1). Spacing between adjacent genes varies from 1,586 bp (between *MtCBF5* and *MtPERLD*) up to 318,549 bp (between *MTR*_*054s0019* and *MTR*_*6g089580*). Coding sequences have an overall G + C content of 42.15%. Sixteen genes are supported by cognate EST and additionally 7 of them by cDNA sequences (Table 1). Comparisons between genomic and EST/cDNA sequences indicate that four genes (i.e. *MtCBF8*, *MtCBF6*, *MtPERLD* and *MTR*_*054s0019*) have 2 transcript isoforms each.

**Table 1:** **Positional candidate genes for Mt-FTQTL6**

Gene ID [reference]	BAC clone and/or WGS sequence	EST (1)	cDNA (1)	Number of splicing isoforms	Number of intron(s) in the coding sequence [intron size(s) in bp]	Putative gene product	Amino acid length [length of the acidic C-terminal domain] (2)	Theoretical molecular weight (KDa)	Theoretical pI [pI of the C-terminal domain] (2)
*MtBAG* **-** *1*[40]	**-**	AW299190; BG646253; AW257319; AW256371; BI308136; AL369679; AW329645; AL373067; BF006595; AW694037; BF650244; AL373068; CA919955; AL373067; CA917144; BE325495; EX533119; BG588510; BE322176	-	1	-	Bcl-2-associated athanogene	355	39.9	9.37
*MtCBF14*	Scaffold065bis	-	-	-	-	CBF/DREB1 protein	230 [103]	25.74	5.55 [4.02]
*MtCBF13*	Scaffold065bis	-	-	-	-	CBF/DREB1 protein	-	-	-
*MtCBF3*[32]/*MtDREB1C*[14]	Scaffold065bis	CA920049; CF069650; BG581707; BF005835; BQ138271; BG582534	DQ267620; EU139868	1	-	CBF/DREB1 protein	227 [109]	25.99	5.64 [4.11]
*MtCBF12*	Scaffold065bis	GT136747; GT141343	BT134401	1	-	CBF/DREB1 protein	-	-	-
*MtCBF11*	Scaffold065bis	-	-	-	-	CBF/DREB1 protein	281 [103]	31.21	5.66 [4.07]
*MtCBF2*[32]	Scaffold065bis; mth2-221P20; mte1-60A22	BF520341	EU139867	1	-	CBF/DREB1 protein	230 [106]	26.07	5.8 [4.12]
*MtCBF10*	Scaffold065bis; mth2-221P20; mte1-60A22	-	-	-	-	CBF/DREB1 protein	198 [104]	22.58	5.39 [4.03]
*MtCBF9*	Scaffold065bis; mte1-60A22	CX528143	-	1	-	CBF/DREB1 protein	208 [114]	24.04	4.99 [3.94]
*MtCBF8* **(a)**	Scaffold065bis; mte1-60A22	*EY477920*; BQ165478; CA921976; *BF005540*; AW573630; BQ165479; EY476564; BE999274; BE998545;BE999273; *CB893700*	DQ778006; *BT146058*	2	no intron/1 intron [316]	CBF/DREB1 protein	215 [100]/271 [156]	24.87/31.11	6.1 [4.27]/8.37 [4.92]
*MtCBF7*	Scaffold065bis; mte1-60A22	BF005905	-	1	1 intron [279]	CBF/DREB1 protein	245 [130]	27.88	6.66 [4.60]
*MtCBF6*	Scaffold065bis; mth2-92O15	CF069139; AW775188; *BF006671*;CA919708	-	2	no intron/1 intron [352]	CBF/DREB1 protein	216 [101]/248 [133]	24.67/28.23	6.31 [4.22]/8.41 [4.84]
*MtCBF5*	Scaffold065bis; mth2-92O15	BG648933; BF005756; CA920720	-	1	1 intron [380]	CBF/DREB1 protein	267 [152]	30.54	8.87 [5.04]
*MtPERLD*	Scaffold065bis; mth2-92O15	BF631847; EV255869; AL373022; CB892925; EY476043; BG449960; BG648146; BF639371; BG648635; CX527766; CX527793; CX527254; BI267056; BF521243; BF642637; CX523947; BE319009; AL373021; BF635170; BM779066; BQ152673; AW736243; *BG646840*	BT051744; BT139597	2	4 introns [2807;93;1008;2220]/ 5 introns [123; 2663; 93;1008;2220]	Per1-like family protein	342	39.48	7.6
*MTR*_*050s0020*[23]	Scaffold065bis; mth2-92O15; mth2-53l24 (GB acc AC229695) **(b)**	BF635955; GT141039; GT136423	-	1	15 introns [2336; 137; 162; 266; 470; 89; 86; 126; 80; 626; 88; 84; 86; 80; 450] **(c)**	ABC transporter B family member	715	78.37	9.07
*MTR*_*050s0019*[23]	Scaffold065bis; mth2-92O15; mth2-53l24 (GB acc AC229695)	EV258193; CX522284	BT147320	1	1 intron [721]	Conserved uncharacterized protein	242	26.45	8.56
*MtZFWD*	Scaffold065bis	CX520203; BE239869	-	1	8 introns [939; 162; 143; 306; 104; 78; 598; 759] **(d)**	Zinc finger CCCH and WD40 domain-containing protein	438	47.29	7.85
*MTR*_*054s0001*[23]	Scaffold065bis; mth2-12e18 (GB acc AC229727)	BQ138417	-	1	11 introns [1352; 982; 1537; 116; 110; > 418; 443; 1495; 27; 139; 627] **(e)**	DNA 3′- phosphoesterase	338	37.66	6.56
*MTR*_*054s0019*[23]	Scaffold065bis; mth2-12e18 (GB acc AC229727)	CB894631; CF068286; DW018377; BG647692; BE316675; EV256045; BF639952; CB893865; EV261994; BE322046; BI263625; AW691341; CX535182; BI265523; BE998615; CA918979; BI266410; CA858657; BF520558; BF637666; BI267231; *AJ503890*	BT051762; BT148189; BT136112	2	5 introns [938; 202; 1366; 511; 864]/ different translation start site and novel intron of 4532 bp	Sorbitol dehydrogenase-like protein	362	39	6.13
*MTR*_*6g089580*[23]	Scaffold065bis; mth2-50m10 (GB acc AC174372)	BG648131; AW685633; AW586532; AI974700; CA920607	-	1	no intron	unknown protein	628	71.8	8.38

(1) Corresponding EST and cDNA were identified through BLAST searches against *M. truncatula* EST and nr/nt databases in GenBank [36] (January 2013), respectively. In case of alternate splicing, the sequence(s) corresponding to the less represented transcript isoform is(are) in italics; (2) similar to [41], the C-terminal domains of MtCBF2-3;5-11;14 were considered as the amino acid sequences that occur after the signature motif “DSAWR” and that up to the last amino acid of the protein; (a) a cDNA sequence corresponding to *MtCBF8* was directly submitted to GenBank [36] by Chen JR, Guo L, Wang H in 2007. The respective locus was referred to as *DREB1A*; (b) In addition to *MTR*_*050s0019* and *MTR*_*050s0020*, 7 other non-transposon genes could be identified on AC229695 but were not considered in this study. In fact, the corresponding genomic region for these latter, spanning 114,402 kb (1-19,425; 23,144-90,266; 147,607-159,557; 185,484-201,386 bp) is completely missing from Scaffold065bis. Because of its size exceeding 200 Kb, AC229695 seems to have resulted from sequencing inserts of at least two BAC clones, one of which did not originate from Mt-FTQTL6-containing region; (c) The intron/exon structure of *MTR*_*050s0020* from *M. truncatula* Mt3.5 genome assembly has been revised and its corresponding coding sequence found to contain 15 introns instead of 16. *M. truncatula* EST and close protein sequences together with EST from other Papilionoideae species and notably a *Pisum sativum L.* sequence generated by RNAseq (Alves Carvalho et al. in preparation) were used for this purpose; (d) As related EST do not fully cover *MtZFWD* coding sequence, intron positions were predicted after multiple alignment with the coding sequences of *Glyma16g32370* and *Glyma09g27300* and EST sequences from *Lathyrus odoratus L.* (GO316201, GO317026, GO319782), *L. japonicus* (FS347722, FS346164, FS360001) and *Phaseolus acutifolius A. Gray* (HO781358); (e) As the EST BQ138417 does not cover the entire coding sequence of *MTR*_*054s0001*, highly similar EST sequences from *L. japonicus* (GO014516), *Vigna unguiculata (L.) Walp.* (FF549909) and *G. max* (AW184979, FK011826) were used for intron location. The positions and sizes of introns 9 and 10 are different from Mt3.5.

All positional candidate genes for Mt-FTQTL6 were found to have highly similar counterparts across a set of dicotyledonous species for which genome sequence information is available (Additional file 7). All, except for homologous genes to *MTR*_*054s0001*, are contained in syntenic blocks exhibiting large gene order conservation with Mt-FTQTL6 (Additional file 7). Three chromosomal segments in *A. thaliana* and in *Solanum lycopersicum* L., two segments in *Populus trichocarpa* (Torr. & Gray) and a single segment in *Vitis vinifera* L. share colinearity with Mt-FTQTL6. Among legumes, two additional *M. truncatula*, six *Glycine max* (L.) Merr. and three *Lotus japonicus* L. regions are colinear with Mt-FTQTL6. Few rearrangements could be noted among conserved regions including gene loss, local duplication or also translocation events (Additional file 7).

### Twelve candidate genes organized in a tandem array belong to the CBF/DREB1 group of the AP2/EREBP transcription factor family

**Figure 3:**
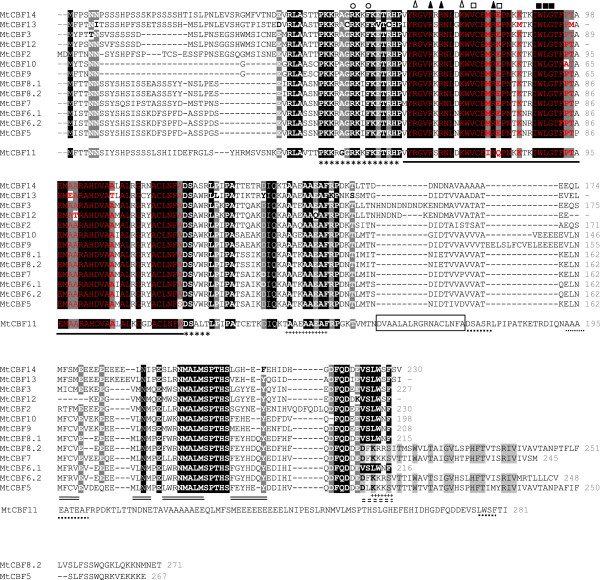
**Alignment of predicted MtCBF2-3;5-14 sequences.** Hypothetical protein sequences are used for *MtCBF12*-*13.* These were obtained after eliminating truncating mutations from the corresponding coding sequences*.* For *MtCBF6* and *MtCBF8*, proteins encoded by both transcript variants are shown. MtCBF11 protein sequence, likely resulting from a chimeric gene, is separated from others. Conserved amino acid residues are in white. They are shaded in black when shared by all sequences or in grey if a single exception exists. Conserved amino acid residues in the C-terminal regions of MtCBF5;6.2;7;8.2 resulting from intron-containing transcripts are in black and grey-shaded. The AP2/ERF domain is underlined and conserved amino acid residues therein are in red. Open squares show conserved valine and glutamic acid residues at the 14th and 19th position of the AP2/ERF domain similarly to other CBF/DREB1 sequences; V14 is critical for determining the DNA binding specificity of CBF/DREB1 proteins [48]. Solid squares indicate the conserved WLG motif in the middle of the AP2/ERF domain which is a common feature with almost all *A. thaliana*, *G. max* and *Oryza sativa* L. genes carrying a single AP2/ERF domain [48, 49]. Solid and open triangles show conserved amino acid residues involved in the interaction of the AP2/ERF domain with target DNA sequences and the sugar phosphate backbone, respectively [50]. The A(A/V)xxA(A/V)xxF [46] and LWSY motifs [47] are underlined with crosses and the CBF/DREB1 signature sequences bracketing the AP2/ERF domain [8] with asterisks. Open circles indicate arginine and phenylalanine residues in the PKK/RPAGRxKFxETRHP signature sequence reported to be critical for DNA binding [44]. The C-terminal clusters of hydrophobic residues [45] that may contribute positively (full lines) or negatively (broken lines) to the trans-activating properties of MtCBF2-3;5-10;12-14 are double-underlined. For MtCBF11, the second (partial) AP2/ERF domain is boxed; the LWSY motif and additional DSAWR and A(A/V)xxA(A/V)xxF motifs are dotted-underlined.

**Figure 4:**
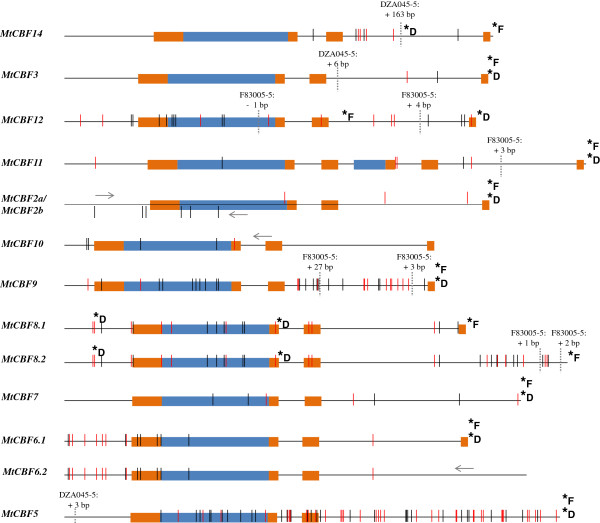
**Polymorphism between F83005-5 and DZA045-5 in the coding sequences of** ***MtCBF2*** **-** ***3*** **;** ***5*** **-** ***12*** **;** ***14*** **.** The order of sequences reflects that of the respective genes in Mt-FTQTL6 region. Schematic representations of the coding sequences of *MtCBF2*-*3*;*5*-*12*;*14* are based on the shortest allele (F83005-5 or DZA045-5) at each time. Blue boxes illustrate regions encoding AP2/ERF domains while orange boxes depict coding regions for characteristic CBF/DREB1 signature sequences, i.e. (from left to right) PKKP/RAGRxKFxETRHP, DSAWR, A(A/V)xxA(A/V)xxF and LWSY. Both alternatively spliced isoforms of *MtCBF6* and *MtCBF8* are shown. *MtCBF2a* and *MtCBF2b*, obtained from F83005-5 using primer sets designed on the same gene sequence from A17, are both compared to the unique sequence obtained from DZA045-5 with these primers. Black and red horizontal lines indicate the positions of synonymous and non-synonymous SNP, respectively. Dotted horizontal lines indicate the positions of indel or SSR polymorphisms. Arrows are used to delimit comparable sequences between F83005-5 and DZA045-5 in case only a partial sequence could be obtained from one or both accessions. Asterisks followed by letters “F” or “D” show the positions of stop codons for F83005-5 and DZA045-5, respectively.

*CBF*/*DREB1* genes represent 60% of all candidates for Mt-FTQTL6 (Table 1). They are organized in a tandem cluster spanning a region of approximately 296 Kb (Figure 2). No intervening spacer genes, except those for retrotransposons, exist in between. *MtCBF12* and *MtCBF13* are pseudogenes. A deletion of a guanine at position 314 in *MtCBF12* coding sequence causes a frameshift and a premature stop codon at the 148^th^ amino acid. *MtCBF13* coding region shows an in-frame 848-bp insertion and two point mutations leading each to a premature stop codon. *MtCBF2*-*3*;*5*-*11*;*14* are likely to represent functional protein-coding genes. The corresponding proteins share 61 to 92% overall amino acid identity (Additional file 8) and all show typical features of the CBF/DREB1 group (Figure 3). They have each a 58-amino acid long AP2/ERF nuclear signaling and DNA binding domain [42–44] and an acidic C-terminal region [isoelectric point (pI) ranging between 3.94 and 5.04] that may function in trans-activation [45]. MtCBF11 contains in addition to the 58-amino acid AP2/ERF domain, a 17-amino acid long sequence most likely corresponding to the C-terminal end of a second AP2/ERF domain (Figures 3 and 4). CBF signature sequences comprising PKKP/RAGRxKFxETRHP and DSAWR motifs bracketing the AP2/ERF domain [8] are largely conserved among MtCBF2-3;5-11;14. These latter also show A(A/V)xxA(A/V)xxF [46] and LWSY motifs [47], reported to be conserved in CBF/DREB1 proteins in different plant species. Variants at the CBF/DREB1 characteristic amino acid sequences are listed in Additional file 9. The (L/Y)(L/Y)x(N/S)(M/L)A(E/Q)G (M/L)(L/M)xxPP sequence, previously suggested as a CBF/DREB1 conserved motif [46] is not present in MtCBF2-3;5-14 except for the NMA motif. These proteins have rather a NM(A/V)LMSPTHS conserved sequence at the same position.

### Distinct levels of polymorphism exist between *MtCBF2-3;5-12;14* alleles from F83005-5 and DZA045-5

*MtCBF2*-*3*;*5*-*12*;*14* specific primers yielded positive amplification from F83005-5 and DZA045-5 indicating that all 11 genes are present in these accessions. No amplification could be obtained with *MtCBF13*-specific primers from both accessions despite using different primer combinations and PCR conditions. For *MtCBF2*, two distinct PCR products were obtained from F83005-5 (named MtCBF2a and MtCBF2b) using distinct forward primers but not from DZA045-5, thus indicating that this gene has undergone duplication at least in F83005-5’s background. Overall, 14,953 bp (F83005-5) and 15,038 bp (DZA045-5) of comparable sequences were generated (Additional file 10). They show a total of 402 single nucleotide polymorphism (SNP), 41 insertion-deletion (indel) and 9 simple sequence repeat (SSR) polymorphisms with 206 SNP, 8 indel and 3 SSR located in the coding sequences (Additional file 10). Contrary to A17 and F83005-5, DZA045-5 has an insertion of a 160-bp short interspersed nuclear element (SINE) in the coding sequence of *MtCBF14* and another SINE element in the 5′ upstream region of *MtCBF8*. F83005-5 has a specific insertion of a putative miniature inverted-repeat transposable element in the 5′ upstream region of *MtCBF6*.

Figure 4 illustrates allelic variation between F83005-5 and DZA045-5 in the coding regions of *MtCBF2*-*3*;*5*-*12*;*14*. As it could be noticed, nucleotide changes leading to differences at the amino acid level are not evenly distributed amongst genes. Amino acid substitution difference between F83005-5 and DZA045-5 ranges between 1 (MtCBF3) and 34 (MtCBF5). *MtCBF14* and *MtCBF8* are likely to encode truncated proteins in freezing-sensitive accession DZA045-5 but not in freezing-tolerant accessions F83005-5 and A17. In contrast, *MtCBF12* is likely to encode a full-length protein in DZA045-5 which is not the case for F83005-5 and A17.

## Discussion

*M. truncatula* is a valuable forage crop and also a prominent model for legume genomics. Freezing tolerance QTL have been previously identified in *M. truncatula*[38]. In the current study, Mt-FTQTL6 has been finely mapped by genetically and physiologically characterizing recombinant haplotypes carrying recombination events within the QTL confidence interval. Genomic data have been developed to fill the genome assembly gap in Mt-FTQTL6 region and have permitted to reveal twenty positional candidate genes. Interestingly, twelve genes belong to the CBF/DREB1 group of the AP2/EREBP transcription factor family and occur in tandem array.

### Current knowledge on the functions of the twenty positional candidate genes for Mt-FTQTL6

The fine mapping step has significantly reduced Mt-FTQTL6′s confidence interval and has subsequently allowed the identification of 20 candidate genes. Functional data regarding these candidate genes are still limited at present (Additional file 11). Only homologs/orthologs of *MtCBF2*-*3*;*5*-*14*, *MtBAG*-*1*, *MTR*_*054s0019* and *MtZDP* have been studied in plant species [13, 15, 35, 51–54]. For *MtPERLD* and *MTR*_*050s0020* which are members of evolutionary conserved gene families, only non-plant homologs have been described [55–57]. *MtZFWD* contains both a CCCH zinc finger domain and WD40 repeats and thus belongs to a plant-specific subgroup of CCCH zinc finger protein family [58] that is not yet functionally described. No data are available for *MTR*_*050s0019* and *MTR*_*6g089580* to infer putative functions. Out of all candidate genes (Table 1), only *MtCBF2* and *MtCBF3* have been functionally characterized in *M. truncatula. MtCBF2* and *MtCBF3* were reported to display both a rapid and transient accumulation of transcripts in the leaves of 3-week old *M. truncatula* plants after exposure to low temperature treatment [32, 34]. Furthermore, transgenic *M. truncatula* lines overexpressing *MtCBF3* were shown to exhibit an improved freezing tolerance compared to the wild-type [14]. However, even if the *CBF*/*DREB1* genes can be considered as the most likely candidates for the Mt-FTQTL6 effect, none of the non-functionally characterized genes can be excluded at present. Further knowledge needs to be gained regarding the different candidate genes and the QTL of interest itself.

### Co-location between a freezing tolerance QTL and *CBF*/*DREB1* genes in a legume species

*CBF*/*DREB1* genes were found to co-locate with QTL for freezing tolerance in several plant species including *A. thaliana*[59, 60], *Triticum monococcum* L. [61], *Triticum aestivum* L. [62] and *Hordeum vulgare* L. subsp. *vulgare*[63]. A large deletion in the promoter region of *A. thaliana CBF2*[59] and co-locations of freezing tolerance QTL in *T. monococcum*[61] and *H. vulgare* subsp. *vulgare*[63] with QTL for expression of cold-induced *CBF/DREB1* target gene *COR14b* and accumulation of COR14b protein suggested *CBF/DREB1* genes being responsible for the QTL effects. The identification of *CBF*/*DREB1* genes among positional candidates of Mt-FTQTL6 provides an additional example of a co-location between a freezing tolerance QTL and this group of genes but for the first time in a legume species. It will be of great interest to inspect such co-location in other legumes especially *P. sativum* having a freezing damage QTL syntenic to Mt-FTQTL6 [39].

### Large size of the CBF/DREB1 group in *M. truncatula*

This study has permitted the physical positioning of twelve *M. truncatula CBF*/*DREB1* genes through the assembly of Mt-FTQTL6 region. Apart from *MtCBF2* and *MtCBF3* that were identified by Pennycooke et al. [32] and *DREB1A* whose cDNA sequence was directly submitted to GenBank (GB acc DQ778006), the other *CBF*/*DREB1* genes associated with Mt-FTQTL6 were not previously annotated or described (Table 1). As the name “MtCBF4” has been recently assigned to a *CBF*/*DREB1* gene located on *M. truncatula* chromosome 1 [35], the novel genes including *DREB1A* were here given consecutive numbers from 5 to 14 starting from the most proximal to *MtPERLD* (Figure 2).

*MtCBF2*-*3*;*5*-*14* occur in tandem array and are in direct orientation (Figure 2) which suggests that they are most likely derived from an unequal crossing over mechanism [64, 65]. However, the contribution of transposable elements to this duplication event cannot be totally excluded [65], especially with several transposon and retrotransposon insertions being evident between *CBF*/*DREB1* genes (data not shown).

According to Young et al. [23], *M. truncatula* has experienced high rates of local gene duplication compared to other plant genomes. It may thus not be surprising to have such a large tandem array organization for the *MtCBF2*-*3*;*5*-*14* locus. Similar tandem duplications have been described for nucleotide-binding site-leucine-rich repeat genes [66] and lipoxygenase genes [67] on *M. truncatula* chromosomes 6 and 8, respectively. However, the main particularity of the tandem cluster in Mt-FTQTL6 region is that it concerns *CBF*/*DREB1* genes. Large groups of *CBF*/*DREB1* genes have always been thought to be limited to monocotyledons. This study reports for the first time that the *CBF*/*DREB1* genes can also be highly duplicated in legume species. *M. truncatula* has at least 17 *CBF*/*DREB1* genes (considering *MtCBF2a* and *MtCBF2b* from F83005-5) all located in homoeologous regions (Additional file 7). A recent report has similarly shown that *Eucalyptus grandis* genome contains 17 *CBF*/*DREB1* genes [68]. It will be next important to determine if other legumes have as many genes that belong to the CBF/DREB1 group as *M. truncatula*. First evidences denote that there may not be a general trend at this level. As shown in Additional file 7, *G. max* does not have any duplication of *CBF*/*DREB1* genes in Mt-FTQTL6 syntenic regions. In contrast, *L. japonicus* has 7 tandemly-arrayed putative *CBF*/*DREB1* genes on chromosome 4.

### Complexity of the CBF/DREB1 locus on *M. truncatula* chromosome 6

As for other duplication modes, tandem duplication generates identical genes that evolve in different ways [65, 69–75]. In the case of the locus harboring *MtCBF2*-*3*;*5*-*14*, some evidences of pseudogenization are apparent: premature stop codons leading to truncated proteins exist within the corresponding coding sequences of *MtCBF13* and *MtCBF12* in A17, *MtCBF12* in F83005-5 and *MtCBF8* and *MtCBF14* in DZA045-5 (Figure 4). However, most *CBF*/*DREB1* duplicates are likely to encode functional proteins. Up to date, information regarding the expression profiles of *MtCBF2*-*3*;*5*-*14* is still greatly lacking. *M. truncatula* gene expression atlas [76] provides only a first glimpse on the expression of some genes among *MtCBF2*-*3*;*5*-*14* under unstressed conditions (Additional file 12). It is thus crucial to conduct a complete survey on the expression patterns (including under cold stress) of all *CBF*/*DREB1* genes on *M. truncatula* chromosome 6. This will determine whether tandemly duplicated *CBF*/*DREB1* genes have evolved or not different expression patterns and so possibly distinct functions as already reported for different *CBF*/*DREB1* genes in other plant species [10, 41, 77, 78]. The characterization of *MtCBF11* deserves a particular attention as its predicted protein product contains a full-length and an additional 17-amino acid long partial AP2/ERF domain (Figure 3 and 4). *MtCBF11* has most likely arisen from an in-frame fusion of the 5′ portion of the coding region from a first *CBF*/*DREB1* gene with the 3′ portion of the coding sequence from a second gene.

Alternative splicing events can diverge between duplicates, including organ- and stress-specific differences, and result in functional variation [79]. Based on genomic sequence alignment with EST and cDNA data, *MtCBF6* and *MtCBF8* have two transcript variants each, generated by intron retention/splicing events (Table 1). *MtCBF5* and *MtCBF7* contain each an intron at the same position as for *MtCBF6* and *MtCBF8* intron-spliced isoforms but no evidence yet suggests that *MtCBF5* and *MtCBF7* may have intron-retaining transcript forms similar to *MtCBF6* and *MtCBF8*. As EST databases can be incomplete with regard to alternative splicing transcript variants, reverse transcription PCR or transcription analysis using second generation sequencing would allow determining alternative splicing patterns with more precision. This is the first study to provide data on the presence of introns and alternative splicing forms in *CBF*/*DREB1* genes. Each of the isoforms of *MtCBF5*-*8* will need to be characterized apart in order to determine if these are functional and then compare them to other *CBF*/*DREB1* genes and alternatively spliced forms if any. Any possible association of alternative splicing of *CBF*/*DREB1* genes with *M. truncatula* response to low temperatures should be inspected. Recent reports from grasses have shown the existence of alternatively spliced isoforms for AP2/EREBP family members [80–82] homologous to *A. thaliana DREB2*[6] which is known to be activated by dehydration and high-salt stresses. The alternative splicing of these genes was suggested to be important for reducing the activity of their target genes under unstressed conditions and thus preventing negative effects on plant growth.

## Conclusions

The present study has permitted to narrow down the confidence interval of a major freezing tolerance QTL on *M. truncatula* chromosome 6 and to identify genes located therein. No similar studies related to legume freezing tolerance have to date been reported. Candidate genes represent important elements toward positional cloning of Mt-FTQTL6. Information gained from *M. truncatula* being a model species should be transferred to agronomically and economically important crop legumes. The BAC clone contig map constructed for the assembly gap coinciding with Mt-FTQTL6 will be interesting in efforts to clone important loci mapped to the same region as Mt-FTQTL6 including a locus for resistance to *Subterranean clover mottle virus*[83] and a QTL for the number of secondary branches [84]. When sequenced from F83005-5 and DZA045-5, *MtCBF2*-*3*;*5*-*12*;*14* coding sequences showed distinct levels of indels and non-synonymous mutations which suggest that they have evolved in a heterogenous manner. Allelic variation identified on coding and immediate flanking sequences of *MtCBF2*-*3*;*5*-*12*;*14* will be useful for future studies to establish the adaptative value of the different *CBF*/*DREB1* duplicates.

## Methods

### Development of plant material

*M. truncatula* LR3 population comprises 178 F_2:5_ recombinant inbred lines (RIL) obtained by single seed descent from a cross between the freezing-tolerant accession F83005-5 and the freezing-sensitive accession DZA045-5 [85]. F83005-5 derives from a natural population collected in France and DZA045-5 from an Algerian population [86]. Genotypic data from the LR3 population [38] indicate that twelve RIL (RIL4, 13, 17, 22, 76, 90, 101, 151, 161, 165 and 241) have residual heterozygosity at Mt-FTQTL6 peak marker, MTIC153. Among them, RIL76 is particularly important because: (1) it is homozygous at the confidence intervals of both other freezing tolerance QTL from the LR3 population mapped on chromosomes 1 and 4 [38]; (2) it carries the sensitive parent’s alleles at both these QTL; and (3) it shows a low overall genome heterozygosity. RIL76 sister lines were therefore chosen for the development of inbred lines showing recombination events at Mt-FTQTL6. Twelve F_5_ lines were grown and submitted to a genotypic analysis using: SSR markers evenly spaced on the eight *M. truncatula* chromosomes (3 to 6 markers per chromosome; 33 markers in total), the nearest SSR marker to the peak of each of the freezing tolerance QTL on chromosomes 1 and 4, and SNP and SSR/indel markers closely linked to Mt-FTQTL6 (6 markers in total). Information regarding Mt-FTQTL6-linked markers is available in Additional file 1. The complete list for the rest of markers is available upon request. Similarly to RIL76, sister lines did not show heterozygosity for any of the background markers and were all homozygous carrying alleles from the freezing-sensitive parent at the freezing tolerance QTL on chromosomes 1 and 4. Five heterozygous recombinants at Mt-FTQTL6 were identified, namely 76-02, 76-05, 76-07, 76-08 and 76-09. Taking advantage of their heterozygosity for all markers associated with Mt-FTQTL6, 76-06 and 76-11 were selected for the construction of large segregating populations. Line 76-02 was also used for this purpose because it is only homozygous for two markers at the upper border of Mt-FTQTL6, namely NT6005 and NT6001 (Figure 1). F_6_ populations that were obtained through self-pollination of 76-02, 76-06 and 76-11 comprised 447, 241 and 232 plants, respectively. Progenies were genotyped using 7 SNP and 6 SSR/indel markers located on both sides of marker MTIC153 (Additional file 1). For recombinant individuals, genotyping was further completed with 13 additional SNP markers (Additional file 1). A high-resolution linkage map constructed from a subset of marker data scored on 76-06 and 76-11 progenies is described in Tayeh et al. [39] (Additional file 2). F_5_ and F_6_ heterozygous recombinant individuals were self-pollinated. Homozygous (F_6_ or F_7_) recombinants were selected from their progenies using corresponding markers. F_7_ and F_8_ plants were finally obtained through the self-pollination of homozygous recombinant plants and were submitted to freezing tolerance tests (Figure 1).

### Plant growth

Seeds of *M. truncatula* were scarified using sand paper in order to rupture the seed coat. Scarified seeds were soaked in distilled water for 6 hours and spread on moistened filter papers in Petri dishes. After a 3-day dark storage at 6°C to break embryo dormancy and synchronize germination, Petri dishes were held at 20°C for three other days. Seedlings were then transplanted in 2 L pots containing a mix of peat and compost or in pre-moistened 41 × 42-mm jiffy-7 pellets (Jiffy France S.A.R.L., Trevoux, France), depending on the experimental need. In case of non-germinated seeds, especially when they were recently harvested, 3 to 5 parts per million of 2-chloroethylphosphonic acid were applied to Petri dishes to break embryo dormancy and seedlings were transplanted 24 hours later. Except for freezing tolerance tests, plants were finally left to grow in the greenhouse at an average temperature of 18/14°C (day/night) and a 16-hour photoperiod. Seeds were extracted from ripe pods using in most cases a homemade extraction system based on a rubber mat and a plaster hawk as described by Garcia et al. [87]. Large extractions were performed using a seed mill.

### Marker development and genotyping

In addition to markers from Tayeh et al. [39], 8 new markers associated with Mt-FTQTL6 were developed in this study (Additional file 1). Primers were designed from BAC-end sequences using the Primer3 tool [88] with default parameters. Maximum product length was limited to 500 bp. Total genomic DNA was isolated from fresh leaflets harvested on one-month-old plants using Nucleospin 96 Plant II (Macherey Nagel, Germany) according to manufacturer’s instructions. Genotyping of the plant material from this study with background and Mt-FTQTL6-associated markers was conducted as described in Tayeh et al. [39]. Capillary gel electrophoresis was employed for SSR and indel markers. The high-resolution melting technique (in case of F_7_/F_8_ lines) and the competitive allele specific PCR method (in case of 447-plant 76-02-derived population) were used for SNP markers.

### Evaluation of freezing tolerance

Freezing tolerance tests were performed in a climate-controlled walk-in 2.6 × 2.3 × 2 m growth chamber. Two experiments, consisting of two replications each, were carried out. Each replicate was composed of: (1) F_7_ or F_8_ plants carrying one (or two) recombination event(s) within Mt-FTQTL6-containing chromosomal region, (2) plants obtained from the same parental lines as the recombinant ones or from sister parental lines but that ideally do not have recombination events within Mt-FTQTL6 region and (3) parental accessions F83005-5 and DZA045-5 (Figure 1). Ten individuals from each of the above-cited lines were evaluated per replicate. Seedlings, transplanted in Jiffy pellets (see plant growth section), were placed in 10 × 10 well-containing homemade Styrodur plates to ensure root insulation. Four different phases were applied during each freezing tolerance test with the following environmental conditions: (1) nursery phase-18 days: 20°C/14°C, 14-hour photoperiod and 250 μmol.m^-2^.s^-1^ photosynthetically active photon flux density (PPFD) provided by fluorescent lights; (2) cold acclimation phase-20 days: 8°C/2°C, 14-hour photoperiod and 250 μmol.m^-2^.s^-1^ PPFD; (3) freezing phase-8 days: 4°C/-6°C, 10-hour photoperiod and 150 μmol.m^-2^.s^-1^ PPFD; and (4) recovery phase-16 days: 16°C/5°C, 14-hour photoperiod and 250 μmol.m^-2^.s^-1^ PPFD. Continuous acquisition of chamber air temperature was carried out using a Campbell CR10x system equipped with a Humitter 50Y integrated humidity and temperature transmitter (Vaisala, Vantaa, Finland). Temperatures at leaf- and root- levels were also continuously surveyed using eight and four homemade T-type thermocouples connected to the acquisition system, respectively. Photosynthetically active radiation was checked twice per test phase by means of a LiCor 190SA quantum sensor (Licor Biosciences, Lincoln, Nebraska, USA). Irrigation was performed manually and stopped 2 days before and all along the freezing phase. Developmental stages were scored plant-by-plant six times during the nursery and cold acclimation phases. At the 16^th^ day of rewarming, plants were evaluated for freezing tolerance using a visual rating scale based on leaf injuries and ranging from 0 (no freezing injuries) to 5 (all leaflets are yellow and dry: dead plant). Plants were scored 1 when less than one quarter of the leaflets were visually damaged, 2 for one quarter of the leaflets being affected, 3 when freezing damage was observed on half of the leaflets and 4 when three-quarter of the leaflets were concerned. Pairwise comparisons of damage scores from recombinant, non-recombinant and parental lines were performed by one-way analysis of variance followed by a Tukey test at P ≤ 0.05 using R [89] (Figure 1). For each of these pairwise comparisons, the recombinant line and its non-recombinant counterpart(s) were compared between each other and with the closest plants of F83005-5 and DZA045-5 within the growth chamber.

### Full BAC sequencing

BAC DNA was isolated using Nucleospin fast purification Kit (Macherey-Nagel, Düren, Germany). Full BAC sequencing was performed using 454 multiplexing technology on a GS-FLX Titanium sequencer (454 Life Sciences, Roche Diagnostics, Branford, Connecticut, USA). Raw reads were cleaned using PyroCleaner [90]. Reads sharing more than 95% homology with BAC vector or *Escherichia coli* genome sequence were excluded. *de novo* assembly of the cleaned reads was performed using Newbler (version 2.3; 454 Life Sciences, Roche Diagnostics). The resulting contigs were, when possible, ordered and oriented based on marker sequences located therein. End sequences from overlapping BAC clones (Additional file 5) were used for the same purpose. Complementarily, end sequences of the different contigs were submitted to blastn/blastx [91] search against *M. truncatula* nr/nt, high-throughput genomic sequence, EST, genome survey sequence and protein databases in GenBank [36] at the National Center for Biotechnology Information. End sequences sharing more than 99% identity with the same target sequence(s) were considered as indicative of adjacent contigs.

### Sequencing of *CBF*/*DREB1* genes from F83005-5 and DZA045-5

The isolation and sequencing of *MtCBF2-3;5-14* from F83005-5 and DZA045-5 was undertaken in order to determine if all genes composing the *CBF*/*DREB1* cluster in A17 are similarly present in these accessions and to identify the polymorphism in the coding and immediate flanking sequences of these genes. Two to 3 specific forward and also 2 to 3 specific reverse primers were designed per gene. PCR were carried out using a touchdown protocol with the following thermal cycling conditions: 4 minutes at 94°C; 5 cycles at 94°C for 30 seconds, annealing temperature (Ta) [-1°C/cycle] for 30 seconds, 72°C for 90 seconds; 35 cycles at 94°C for 30 seconds, [Ta-5°C] for 30 seconds, 72°C for 90 seconds; and 10 minutes at 72°C. Different primer combinations and PCR conditions (Ta and/or MgCl_2_ concentration) were tested to optimize the amplification result. PCR products from the optimal conditions were purified from agarose gel using Nucleospin Extract II (Macherey-Nagel, Düren, Germany) following manufacturer’s instructions and Sanger-sequenced using BigDye Terminator v3.1 chemistry (Applied Biosystems, Foster City, California, USA) on a 3130×l Genetic Analyzer (Hitachi/Applied Biosystems). Primers and amplification conditions that were used in the sequencing step are provided in Additional file 10. For 9 out of the 12 *CBF*/*DREB1* genes, one or two internal primers were needed to ensure full sequence coverage and/or overcome sequencing difficulties caused by simple sequence repeats [(A)_n_ or (T)_n_] present in 5′- or 3′-non-coding flanking regions (Additional file 10). Base calling was performed with Sequencing Analysis Software (version 5.4; Applied Biosystems). Electropherograms were manually trimmed of poor sequence data.

### Availability of supporting data

The data sets supporting the results of this article are included within the article and its additional files. All sequences were submitted to GenBank [36]. Insert sequences of BAC clones mth2-92O15, mte1-60A22 and mth2-221P20 were deposited in the “high throughput genomic sequences” division under accession numbers KF006382-84. BAC-end sequences were submitted to the “genome survey sequences” division and have been assigned the accession numbers JY974377-472. *CBF*/*DREB1* gene sequences from A17, F83005-5 and DZA045-5 were deposited in the “plant, fungal, and algal sequences” division with accession numbers KC997199-225.

## Electronic supplementary material

Additional file 1: **List of markers used for plant material development and BAC clone contig map construction.** Provides a list of the markers that were used for one or more of the following purposes: (1) genotyping of F_6_ segregating populations; (2) genotyping of F_7_/F_8_ lines used for Mt-FTQTL6 fine mapping; (3) synthesis of the PCR products used as probes for BAC filter screening and (4) validation of positive BAC clones and BAC contig construction. It includes primer sequences, PCR conditions and information on the type of polymorphism for corresponding markers. (XLS 42 KB)

Additional file 2: **Genetic linkage map of Mt-FTQTL6 region.** Contains a partial linkage map of *M. truncatula* chromosome 6 which was constructed using data collected on 453 F_6_ plants. It shows the relative positions of the different markers linked to Mt-FTQTL6 including MTIC153 and NT6054 that border its confidence interval. (DOC 34 KB)

Additional file 3: **Construction of a BAC clone contig map spanning Mt-FTQTL6-containing region.** Describes the strategy that was followed to close the physical gap coinciding with Mt-FTQTL6 and summarizes the main results. The text is organized in three sections: (1) State of art; (2) Methods and (3) Achievements. (DOC 62 KB)

Additional file 4: **Positive PCR-confirmed BAC clones obtained through screening of high-density colony filters with 15 probes.** Lists the BAC clones from *M. truncatula* mth2 and Mtf83 libraries that were identified by screening high-density colony filters with probes corresponding to 15 Mt-FTQTL6-linked markers and were further confirmed positive by PCR control. (XLS 34 KB)

Additional file 5: **BAC clone contigs partly spanning** ***M. truncatula*** **chromosome 6 region harboring Mt-FTQTL6.** Depicts the six BAC clone contigs that were constructed with BAC clones identified either through screening of *M. truncatula* BAC libraries mth2 and Mtf83 (see Additional file 4) or using an *in silico* approach. The BAC clone contig map is connected to the genetic map of Mt-FTQTL6 by means of the 15 markers used as screening probes and other additional markers. The three BAC clones that were selected for insert sequencing are shown. (XLS 88 KB)

Additional file 6: **Statistics of 454 BAC sequencing and contig assembly.** Contains information relative to 454 sequencing of BAC clones mth2-92O15, mte1-60A22 and mth2-221P20. Statistics on raw, cleaned and assembled sequences are given. (XLS 36 KB)

Additional file 7: **Colinearity conservation between Mt-FTQTL6 interval and chromosomal regions from sequenced dicotyledonous genomes.** Illustrates colinearity conservation between Mt-FTQTL6 region and different genomic segments from reference dicotyledonous plant species including *M. truncatula*, *G. max*, *L. japonicus*, *P. trichocarpa*, *A. thaliana*, *V. vinifera* and *S. lycopersicum.* (XLS 38 KB)

Additional file 8: **Pairwise comparison of predicted protein products of** ***MtCBF2*** **-** ***3*** **;** ***5*** **-** ***10*** **;** ***12-*** ***14.*** Shows amino acid identity and similarity between the predicted protein products of *MtCBF2-3;5-10;12-14.* This information is important to understand the phylogenetic relationships between tandemly duplicated *CBF/DREB1* genes. (XLS 30 KB)

Additional file 9: **Amino acid variants within CBF/DREB1 signature sequences.** Reports the amino acid variants in conserved CBF/DREB1 signature motifs PKKP/RAGRxKFxETRHP, DSAWR, A(A/V)xxA(A/V)xxF and LWSY that are carried by MtCBF2-3;5-11;14. Other CBF/DREB1 proteins sharing the same amino acid variant(s) are indicated when possible. (XLS 36 KB)

Additional file 10: **Sequencing of** ***MtCBF2-3;5-12;14*** **: primers, PCR conditions and sequence analyses.** Contains details on the primers that were used to isolate and sequence *MtCBF2-3;5-12;14* from F83005-5 and DZA045-5. For each of the *CBF/DREB1* genes, SNP, indel and SSR polymorphisms identified between F83005-5 and DZA045-5 together with amino acid variations amongst the predicted protein products are indicated. (XLS 52 KB)

Additional file 11: **Bibliographic review regarding non-** ***CBF/DREB1*** **candidate genes for Mt-FTQTL6.** Briefly reviews the current knowledge on non-*CBF/DREB1*positional candidate genes for Mt-FTQTL6 and particularly their functions. Information is available for *MtBAG-1*, *MtPERLD*, *MTR_050s0020, MtZFWD*, *MTR_054s0001* and *MTR_054s0019* whereas no data could be obtained for *MTR_050s0019* and *MTR_6g089580*. (DOC 57 KB)

Additional file 12: **Expression profiles of a subset of** ***M. truncatula CBF/DREB1*** **genes according to the Gene Expression Atlas.** Shows the mean transcript levels from three independent biological replicates of *MtCBF2*, *MtCBF3*, *MtCBF5*, *MtCBF6*, *MtCBF7*, *MtCBF8.1*, *MtCBF8.2* and *MtCBF9* in four unstressed organs (root, stem, leaf and flower) of the A17 accession. Data were obtained from *M. truncatula* Gene Expression Atlas version 2 [76]. (DOC 60 KB)

## Abbreviations

BAC: Bacterial-artificial chromosome
CBF: C-repeat binding factor
DREB1: Dehydration-responsive element binding factor 1
QTL: Quantitative trait locus
WGS: Whole-genome shotgun
GBacc: GenBank accession number
FDS: Freezing damage score
nr/nt: Non-redundant nucleotide
EST: Expressed sequence tag
cDNA: Complementary DNA
SNP: Single nucleotide polymorphism
indel: Insertion-deletion
SSR: Simple sequence repeat
pI: Isoelectric point
SINE: Short interspersed nuclear element
RIL: Recombinant inbred line
PPFD: Photosynthetically active photon flux density
Ta: Annealing temperature.
