# Supplementary material for: A tandem array of CBF/DREB1 genes is located in a major freezing tolerance QTL region on Medicago truncatula chromosome 6
Source: BMC Genomics. 2013 Nov 21;14(1):814. doi: 10.1186/1471-2164-14-814 (PMC4046650; doi:10.1186/1471-2164-14-814)
Supplement: Supplementary file 2 — Additional file 2: Genetic linkage map of Mt-FTQTL6 region. Contains a partial linkage map of M. truncatula chromosome 6 which was constructed using data collected on 453 F6 plants. It shows the relative positions of the different markers linked to Mt-FTQTL6 including MTIC153 and NT6054 that border its confidence interval. (DOC 34 KB) [file 12864_2013_5512_MOESM2_ESM.doc]

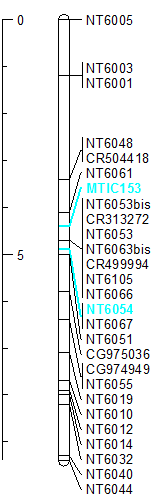


**Additional file 2. Genetic linkage map of Mt-FTQTL6 region**
The linkage map was constructed using genotypic data collected on 453 *M. truncatula* F_6_ plants derived from the self-pollination of two residual heterozygous lines: 76-06 (236 plants) and 76-11 (217 plants). The order of markers was estimated using JoinMap 4.1 [S1] with the regression mapping algorithm (default parameters, recombination frequency < 0.4, logarithm of odds > 2.0) and the Kosambi mapping function [S2]. Ten markers were here added to the original map reported in [39] (see Additional file 1). Markers in blue refer to the markers bordering Mt-FTQTL6 confidence interval (see Figure 1).

Supplementary references:

S1. Van Ooijen JW: **Joinmap® 4, software for the calculation of genetic linkage maps in experimental populations.** Kyazma B.V., Wageningen, Netherlands; 2006.
S2. Kosambi DD: **The estimation of map distances from recombination values.** *Ann Eugen* 1944, **12**:172-175.
