# Supplementary material for: A tandem array of CBF/DREB1 genes is located in a major freezing tolerance QTL region on Medicago truncatula chromosome 6
Source: BMC Genomics. 2013 Nov 21;14(1):814. doi: 10.1186/1471-2164-14-814 (PMC4046650; doi:10.1186/1471-2164-14-814)
Supplement: Supplementary file 3 — Additional file 3: Construction of a BAC clone contig map spanning Mt-FTQTL6-containing region. Describes the strategy that was followed to close the physical gap coinciding with Mt-FTQTL6 and summarizes the main results. The text is organized in three sections: (1) State of art; (2) Methods and (3) Achievements. (DOC 62 KB) [file 12864_2013_5512_MOESM3_ESM.doc]

**Additional file 3. Construction of a BAC clone contig map spanning Mt-FTQTL6- containing region**

a- State of art

In a previous study, Tayeh et al. [39] have found that Mt-FTQTL6 is located between markers NT6001 (BAC clone mth2-156d20; GB acc AC157779) and NT6019 (BAC clone mth2-48m20; GB acc AC225497) which corresponds to an assembly gap on *M. truncatula* chromosome 6 pseudomolecule. Six sequenced BAC clones could be associated to the QTL confidence interval because of close markers to Mt-FTQTL6 being located therein or in their contiguous BAC clones. The aims of this work were to: (1) identify further BAC clones originating from the target chromosomal region; (2) construct a BAC clone contig map encompassing it; and (3) provide a glimpse about the content of this region.

b- Methods for the identification of positive BAC clones, contig assembly and BAC-end sequence analysis

b.1- Preparation of high-density colony filters

BAC clones from *M. truncatula* BAC libraries, mth2 and Mtf83 [23], were spotted onto 22 x 22 cm Immobilon-NY+ filters (Millipore Corporate, Billerica, Massachusetts, USA) using a robotic workstation QPix2 XT (Genetix, Hampshire, UK). mth2 and Mtf83 were developed from the accessions A17 and F83005-5, respectively. Two high-density colony filters were prepared for each of the libraries using either a 7 x 7 (mth2) or a 5 x 5 pattern (Mtf83). Overall, 55,296 unique clones were spotted in duplicate on each filter for the mth2 library, and 27,648 clones for the Mtf83 library. Clones were grown at 37 °C for 17 hours. Filters were then processed as follows: (1) denaturation on Whatman paper soaked with a solution of 0.5 M NaOH and 1.5 M NaCl for 4 minutes at room temperature and for 10 more minutes at 100 °C; (2) neutralization on Whatman paper soaked with 1 M Tris-HCl pH 7.4 and 1.5 M NaCl for 10 minutes, incubation in a solution of 0.25 mg/mL of proteinase K (Sigma Aldrich, St. Louis, Missouri) at 37 °C for 45 minutes, baking at 80 °C for 45 minutes; and (3) fixation by UV on a Biolink 254 nm crosslinker (Thermo Fischer Scientific, Waltham, Massachusetts) with an energy of 120,000 μJoules.

b.2- Colony filter hybridization

After PCR amplification of markers to be used as probes (Additional file 4), the resulting products were purified with Nucleospin Extract II (Macherey-Nagel, Düren, Germany) following manufacturer’s instructions. The elution step was repeated twice in a final volume of 60 µL of ultrapure water. Purified products were run on agarose gel electrophoresis to check for product quality and ensure having at least 10 ng/µL. Probe labelling with [33P]dCTP was performed by random priming using the Ready-To-Go DNA Labelling Beads kit (GE Healthcare, Little Chalfont, UK). One hundred fifty ng of probe DNA were denatured by heating at 95 °C for 10 minutes and incubated with 50 µCi of [33P]dCTP at 37 °C for 4 hours. Unincorporated nucleotides were removed using Illustra ProbeQuant G-50 Micro Columns (GE Healthcare).
Labelled probes were used either in pools or individually to hybridize colony filters. One or both filters of each of the BAC libraries were used. Hybridization of the filters was performed as described in [S3]. Filters were then imaged with a Storm 860 PhosphorImager (GE Healthcare). Analyses were conducted using the HDFR software (Incogen, Williamsburg, Virginia, USA). Positive clones were controlled by PCR tests using the primer sets that initially served for probe synthesis. Concerning the Mtf83 library, only positive BAC clones for markers MTIC153 and NT6047 are listed in this work (Additional file 4).

b.3- BAC-end sequencing

Fifty-seven hybridization-positive BAC clones did not have their BAC-end sequences available neither in the GSS database in GenBank nor in any other public database. They were thus locally sequenced with the following primers: T7, 5’-TAATACGACTCACTATAGGG-3´ and M13, 5´-CAGGAAACAGCTATGACC-3´. Amplification reactions were performed in a final volume of 12 µL containing 200 ng of BAC DNA as template and under a three-step thermal cycling protocol with an annealing temperature of 60 °C and an elongation of 4 minutes. Sequencing was conducted as described in methods for the sequencing of *CBF/DREB1* genes from F83005-5 and DZA045-5. The resulting sequences were submitted to Genbank [36] under the accession numbers JY974377-472.

b.3*- In silico* search for positive BAC clones

*M. truncatula* GSS database was queried using sequences from markers associated to Mt-FTQTL6 and BAC-end sequences from hybridization-positive clones. Only non repetitive hits sharing 99 to 100 % identity with submitted queries were considered positive and their corresponding BAC clones assumed as potentially originating from Mt-FTQTL6-containing region. *in silico* search for positive BAC clones was similarly pursued using for the next round(s) end-sequences from positive clones identified through the previous one and that until no significant hits could be detected. Positive BAC clones were from three BAC libraries (mth2, mth4 and mte1), all obtained from the reference accession A17 [23]. A subset of these clones was validated by PCR.

b.4- BAC clone contig assembly

In order to determine the relative positions of positive BAC clones, end sequences originating from clones found to be positive for the same probes or for probes corresponding to adjacent markers were first aligned against each other using Blast2seq [S4]. They were at the same time aligned against inserts from the first seed BAC clones anchored to Mt-FTQTL6 confidence interval (clone name/GB acc: mth2-154j21/AC146807, mth2-53l24/AC229695, mth2-12e18/AC229727, mth2-15l5/AC130804, mth2-172p22/AC146818, mth2-50m10/AC174372; [39]). To expand data obtained by sequence comparisons, amplification tests were conducted using primers corresponding to different Mt-FTQTL6-associated markers. Some primers were also designed on non-repetitive BAC-end sequences and used for the same purpose. All primers used to control positive BAC clones and to achieve BAC contig construction are provided in Additional file 1.

b.5- BAC-end sequence annotation

Besides being useful for BAC clone contig map construction and genetic marker development, BAC-end sequences can provide information concerning gene and transposable element content of an unsequenced genomic region and allow interspecies physical map comparative studies. This method has been applied for genome-wide analyses [S5,S6]. Only BAC-ends that do not overlap with available full BAC sequences were considered for annotation. BAC-end sequences were assembled into consensi either manually or using CAP3 tool [S7]. Blast searches were conducted against *M. truncatula* nr/nt and HTGS databases at GenBank to identify repetitive sequences. Comparisons between putative repetitive BAC-end sequences and full-length Long-Terminal Repeat (LTR) retrotransposons identified by Wang and Liu [S8] were conducted using Blast2Seq. Hits were considered positive when counterpart sequences showed an identity > 80 %. Sequences were then annotated according to the classification proposed by these authors. Other repetitive sequences were, when possible, annotated after homology search with repetitive sequences from different plant species using Blastn [91] and CENSOR [S9]. Blastx and blastn against plant protein and EST sequences were performed in order to uncover BAC-ends located in genic regions.

c- Achievements of this work

Eleven gene- and four BAC-end sequence-based markers were used as probes to identify positive BAC clones originating from Mt-FTQTL6 region by BAC colony filter hybridization. One hundred six PCR-confirmed clones could be obtained (Additional file 4). Twelve additional positive BAC clones were identified following a blast-based (*in silico*) strategy. Attempts here described did not manage to fully close Mt-FTQTL6 corresponding assembly. BAC clones were assembled into 6 primary contigs.
Inserts from the six sequenced BAC clones previously associated with Mt-FTQTL6 are characterized by a low gene density and high transposon content. However, as shown in Additional file 5, these clones belong to two contigs, viz. I and IV, partly spanning the target chromosomal interval. In order to offer a wider overview of the gene and repetitive element content of Mt-FTQTL6-containing region, 124 non-redundant consensus BAC-end sequences (175 to 1,932 bp individual sequence length; 82,863 bp total length with 36.2% G + C content) from the different contigs were analyzed. Eighty-nine of them (71.77 %) correspond to repetitive sequences in the *M. truncatula* genome, 18 others (14.5%) have likely originated from 13 protein-encoding genes while the rest show no homology to known sequences.
Sixty-three (33 Gypsy; 13 Copia; 17 uncategorized) BAC-end sequences show similarity to LTR retrotransposons; forty-three among them being highly similar to full-length *M. truncatula* LTR elements previously reported [S8]. Seven sequences could be annotated as non LTR-retrotransposons and three others as DNA transposons. Based on high number of blast hits with the *M. truncatula* genome, 16 BAC-end sequences were classified as repeat sequences but could not be annotated.
Non-transposon protein-encoding genes that could be identified through BAC-end sequence analysis are likely to encode: a homeobox-leucine zipper protein (GB acc CR504418), a rhomboid family protein (GB acc CG955596/CG960582), an ABC transporter C family member (GB acc JY974385/CG966926), an IQ-domain containing protein (GB acc CR504417/CG952359), a DNA-directed RNA polymerase II subunit (GB acc JY974388), three consecutive CBF/DREB1 proteins (GB acc CR296231, CR331193 and CG949090), a Per1-like family protein (GB acc CG963227/CG924736), a CCCH Zinc finger motif and WD40 repeat-containing protein (GB acc JY974423), a carboxylesterase-like protein (GB acc CR483596), a protein kinase family member (GB acc CG975036) and a RNA helicase (GB acc CG974949/CG940758). The order of these genes, as it could be determined from the order of corresponding BAC clones, supports high colinearity between Mt-FTQTL6-containing region and orthologous segments *G. max* chromosomes 9 and 16 observed in [39]. However, three genes have their most similar sequences in *G. max* absent either simply on Mt-FTQTL6 co-orthologous (chromosomes 9 and 16) or on any other homologous (chromosomes 5, 10, 17 and 20) block indicating local interruptions of synteny. The likely higher number of *CBF/DREB1* genes in *M. truncatula* compared to all *G. max* homologous regions represent another type of synteny interruption.

Supplementary references:

S3. Sambrook J, Russel DW: Hybridization of bacterial DNA on filters. In Molecular cloning: a laboratory manual. 3rd edition. New York: Cold Spring Harbor Laboratory Press; 2001:138-142.

S4. Tatusova TA, Madden TL: **BLAST 2 Sequences, a new tool for comparing protein and nucleotide sequences.** *FEMS Microbiol Lett* 1999, **174:**247-250.

S5. Gao LL, Hane JK, Kamphuis LG, Foley R, Shi BJ, Atkins CA, Singh KB: **Development of genomic resources for the narrow-leafed lupin (*Lupinus angustifolius*): construction of a bacterial artificial chromosome (BAC) library and BAC-end sequencing.** *BMC Genomics* 2011, **12**:521.

S6. Rampant PF, Lesur I, Boussardon C, Bitton F, Martin-Magniette ML, Bodénès C, Le Provost G, Bergès H, Fluch S, Kremer A, Plomion C: **Analysis of BAC end sequences in oak, a keystone forest tree species, providing insight into the composition of its genome.** *BMC Genomics* 2011, **12**:292.

S7. Huang X, Madan A: **CAP3: A DNA sequence assembly program.** *Genome Res* 1999, **9**:868-877.

S8. Wang H, Liu JS: **LTR retrotransposon landscape in *Medicago truncatula*: more rapid removal than in rice.** *BMC Genomics* 2008, **9**:382.

S9. Kohany O, Gentles AJ, Hankus L, Jurka J: **Annotation, submission and screening of repetitive elements in Repbase: RepbaseSubmitter and Censor.** *BMC Bioinformatics* 2006, **7**:474.
