# Supplementary material for: A tandem array of CBF/DREB1 genes is located in a major freezing tolerance QTL region on Medicago truncatula chromosome 6
Source: BMC Genomics. 2013 Nov 21;14(1):814. doi: 10.1186/1471-2164-14-814 (PMC4046650; doi:10.1186/1471-2164-14-814)
Supplement: Supplementary file 11 — Additional file 11: Bibliographic review regarding non- CBF/DREB1 candidate genes for Mt-FTQTL6. Briefly reviews the current knowledge on non-CBF/DREB1positional candidate genes for Mt-FTQTL6 and particularly their functions. Information is available for MtBAG-1, MtPERLD, MTR_050s0020, MtZFWD, MTR_054s0001 and MTR_054s0019 whereas no data could be obtained for MTR_050s0019 and MTR_6g089580. (DOC 57 KB) [file 12864_2013_5512_MOESM11_ESM.doc]

**Additional file 11. Bibliographic review regarding non-*CBF/DREB1* candidate genes for Mt-FTQTL6**

MtBAG-1 belongs to the evolutionary conserved Bcl-2-associated athanogene (BAG) protein family [S13]. Members of this family all share a conserved C-terminal region that interacts with the ATPase domain of the molecular chaperone 70-kDa heat shock protein (Hsp70/Hsc70) [40]. Amino acid residues required for the interaction of the BAG domain with Hsp70/Hsc70 [51] are conserved in MtBAG-1 (Glu192, Asp202, Arg214 and Gln222) suggesting that it may be a functional protein. Three distinct classes of BAG proteins were identified in *A. thaliana*. MtBAG-1is most similar to proteins from class I (AtBAG1-4; [40]) that carry, in addition to the BAG domain, an N-terminal ubiquitin-like amino acid sequence suggested to interact with the 26S proteasome thus permitting a physical link between Hsp70/Hsc70 and the proteasome. AtBAG4 is the only BAG class I protein that has been functionally characterized in *A. thaliana* [51]. It was reported to be involved in the control of plant growth and development. Knock-out lines were found to senesce earlier than wild-type. AtBAG4 was also shown to be involved in the response to low-temperature stress. A rapid induction of *AtBAG4* was reported 20 minutes after *A. thaliana* lines were exposed to cold stress. Furthermore, contrary to the wild type, the leaves of low level *AtBAG4*-expressing transgenic tobacco lines did not exhibit any cold injury following cold stress (-20°C for 10 minutes) and displayed ~2-fold higher chlorophyll content [51].

MtPERLD shares 26 % identity with *Saccharomyces cerevisiae* Per1 and 28% identity with *Homo sapiens* PERLD1*.* Per1 and PERLD1 are functional homologs sharing 28 % identity [55]. Per1 is involved in the lipid remodeling of the Glycosylphoshatidylinositol-anchored proteins which is crucial for the efficient transport of such proteins from the endoplasmic reticulum to the Golgi apparatus and for their association with lipid rafts. Glycosylphoshatidylinositol-anchored proteins are not correctly localized at the cell surface in *per1* mutant cells [55]. Both histidine residues (H177 and H326), reported to be critical for the function of Per1, are conserved in MtPERLD. To our knowledge, no information is available in the literature regarding the response of *PER1*-like genes to low-temperature stress. Instead, *PER1*-like genes were found to be induced in response to dehydration [S14] or short ethylene treatment [S15] in *A. thaliana*, heat and combined heat and drought stress in *Triticum turgidum* subsp. *durum* [S16] and infection with *Clavobacter michiganensis* subsp. *Michiganensis* in *Solanum lypcopersicum* [S17].

*MTR_050s0020* most likely encodes a member of the *M. truncatula* ATP-binding cassette (ABC) protein superfamily. According to the unified nomenclature proposed by [S18], MTR_050s0020 would belong to the ABC subfamily B, being presumably a half-size TAP (Transporter associated with antigen processing) protein. Half-size TAP proteins have been associated with peptide transport in mammals. Mammalian TAP1 and TAP2 are essential for antigenic peptide presentation on the cell surface [57] [S19,S20]. Three half-size TAP proteins of subfamily B have been identified both in *A. thaliana* and *Oryza sativa* [57] [S18]. MTR_050s0020 is most similar to At4g25450 and Os07g28090. These TAP proteins or other with high identity to MTR_050s0020 have not yet been characterized at the functional level.

MtZFWD is characterized by the presence of an N terminal CCCH zinc finger motif followed by seven putative WD40 repeat domains. CCCH proteins are suggested to have RNA binding abilities [S21]. WD40 repeats are involved in protein-protein interaction [S22]. Four genes from *A. thaliana* [58] [S21,S22], 2 genes from *O. sativa* [S21,S23], 2 genes from *Populus trichocarpa* [S24] encode for proteins containing both CCCH zinc finger and WD40 repeats. *MtZFWD* and its homologs appear to be plant-specific [58]. To our knowledge, the function of these genes is still unknown. The most similar gene to *MtZFWD* from *A. thaliana*, *ZFWD1* (*At4g25440*), was reported to have a xylem-biased expression pattern [S25].

*MTR_054s0001* shares high sequence similarity with Mg2+-dependent DNA 3’- phosphoesterase from *Zea mays* (*ZmDP2*; [53]) and *A. thaliana* (*AtZDP*; [54]). These enzymes were found to act as repair catalysts during oxidative DNA damage being involved in the removal of 3’-phosphate-blocking groups from DNA single-strand breaks in order to enable subsequent gap filling and ligation by dedicated DNA polymerases and ligases. Single-strand breaks represent a blocking obstacle for the replication fork and for the transcription machinery [S26]. They often result from attack to DNA sugar residues by reactive oxygen species produced by the aerobic metabolism taking place in chloroplasts, mitochondria and peroxisomes. As for AtZDP and ZmDP2, MTR_054s0001 contains C-terminal amino acid sequences resembling consensus phosphohydrolase motifs [53]. It also has a poly-(ADP-ribose) polymerase-like zinc finger (CX2CX28HX2C) DNA-binding domain at the N-terminal region indicating that it may act as a DNA nick sensor [54]. Any possible role for DNA 3’- phosphoesterases in response to cold stress still await discovery.

*MTR_054s0019* appears to be a low or single copy number gene in the genome of *M. truncatula*. MTR_054s0019 shares 82% identity with At5g51970. In a recent study, Nosarzewski et al. [52] have reported that At5g51970 acts as sorbitol-metabolizing enzyme. *At5g51970* transcript levels and protein activity peaked early during the drought stress recovery and were associated with a decrease in sorbitol concentration. At5g51970 knockout plants were unable to recover from drought stress after rewatering. They were defective in metabolizing sorbitol and/or ribitol accumulated during drought stress. Sorbitol is a six-carbon sugar alcohol. It is used as a primary translocated photosynthate in many important crops within the Rosaceae. Few reports have to date provided evidence for soritol being involved in response to abiotic stresses [S27 and references cited therein]. No link between sorbitol accumulation and freezing tolerance in legumes has been established.

Supplementary references:

S13. Kabbage M, Dickman MB: **The BAG proteins: a ubiquitous family of chaperone regulators.** *Cell Mol Life Sci* 2008, **65**:1390-1402.

S14. Maruyama K, Takeda M, Kidokoro S, Yamada K, Sakuma Y, Urano K, Fujita M, Yoshiwara K, Matsukura S, Morishita Y, Sasaki R, Suzuki H, Saito K, Shibata D, Shinozaki K, Yamaguchi-Shinozaki K: **Metabolic pathways involved in cold acclimation identified by integrated analysis of metabolites and transcripts regulated by *DREB1A* and *DREB2A*.** *Plant Physiol* 2009*,* **150**:1972-1980.

S15. De Paepe A, Vuylsteke M, Van Hummelen P, Zabeau M, Van Der Straeten D: **Transcriptional profiling by cDNA-AFLP and microarray analysis reveals novel insights into the early response to ethylene in *Arabidopsis*.** *Plant J* 2004, **39**:537-559.

S16. Rampino P, Mita G, Fasano P, Borrelli GM, Aprile A, Dalessandro G, De Bellis L, Perrotta C: **Novel durum wheat genes up-regulated in response to a combination of heat and drought stress.** *Plant Physiol Biochem* 2012*,* **56**:72-78.

S17. Lara-Ávila JP, Isordia-Jasso MI, Castillo-Collazo R, Simpson J, Alpuche-Solís AG: **Gene expression analysis during interaction of tomato and related wild species with *Clavibacter michiganensis* subsp. *michiganensis****. Plant Mol Biol Rep* 2012*,* **30**:498-511.

S18. Verrier PJ, Bird D, Burla B, Dassa E, Forestier C, Geisler M, Klein M, Kolukisaoglu Ü, Lee Y, Martinoia E, Murphy A, Rea PA, Samuels L, Schulz B, Spalding EP, Yazaki K, Theodoulou FL: **Plant ABC proteins – a unified nomenclature and updated inventory.** *Trends Plant Sci* 2008, **13**:151-159.

S19. Sánchez-Fernández R, Emyr Davies TG, Coleman JOD, Rea PA: **The *Arabidopsis thaliana* ABC protein superfamily, a complete inventory.** *J Biol Chem* 2001, **276**:30231-30244.

S20. Sugiyama A, Shitan N, Sato S, Nakamura Y, Tabata S, Yazaki K: **Genome-wide analysis of ATP-binding cassette (ABC) proteins in a model legume plant, *Lotus japonicus*: comparison with *Arabidopsis* ABC protein family.** *DNA Res* 2006, **13**:205-228.

S21. Wang D, Guo Y, Wu C, Yang G, Li Y, Zheng C: **Genome-wide analysis of CCCH zinc finger family in Arabidopsis** **and rice.** *BMC Genomics* 2008, **9**:44.

S22. van Nocker S, Ludwig P: **The WD-repeat protein superfamily in Arabidopsis: conservation and divergence in structure and function.** *BMC Genomics* 2003, **4**:50.

S23. Ouyang Y, Huang X, Lu Z, Yao J: **Genomic survey, expression profile and co-expression network**

**analysis of OsWD40 family in rice.** *BMC Genomics* 2012, **13**:100.

S24. Chai G, Hu R, Zhang D, Qi G, Zuo R, Cao Y, Chen P, Kong Y, Zhou G: **Comprehensive analysis of CCCH zinc finger family in poplar (Populus trichocarpa).** *BMC Genomics* 2012, **13**:253.

S25. Zhao C, Craig JC, Petzold HE, Dickerman AW, Beers EP: **The xylem and phloem transcriptomes from secondary tissues of the Arabidopsis root-hypocotyl**. *Plant Physiol* 2005, **138**:803-818.

S26. Roldán-Arjona T, Ariza RR: **Repair and tolerance of oxidative DNA damage in plants.** *Mutation Res*

2009, **681**:169-179.

S27. Pinho dos Reis S, Medeiros Lima A, Batista de Souza CR: **Recent molecular advances on downstream plant responses to abiotic stress.** *Int J Mol Sci* 2012, **13**:8628-8647.
