# Supplementary material for: A tandem array of CBF/DREB1 genes is located in a major freezing tolerance QTL region on Medicago truncatula chromosome 6
Source: BMC Genomics. 2013 Nov 21;14(1):814. doi: 10.1186/1471-2164-14-814 (PMC4046650; doi:10.1186/1471-2164-14-814)
Supplement: Supplementary file 12 — Additional file 12: Expression profiles of a subset of M. truncatula CBF/DREB1 genes according to the Gene Expression Atlas. Shows the mean transcript levels from three independent biological replicates of MtCBF2, MtCBF3, MtCBF5, MtCBF6, MtCBF7, MtCBF8.1, MtCBF8.2 and MtCBF9 in four unstressed organs (root, stem, leaf and flower) of the A17 accession. Data were obtained from M. truncatula Gene Expression Atlas version 2 [76]. (DOC 60 KB) [file 12864_2013_5512_MOESM12_ESM.doc]

**Additional file 12. Expression profiles of a subset of *M. truncatula* *CBF/DREB1* genes according to the Gene Expression Atlas**


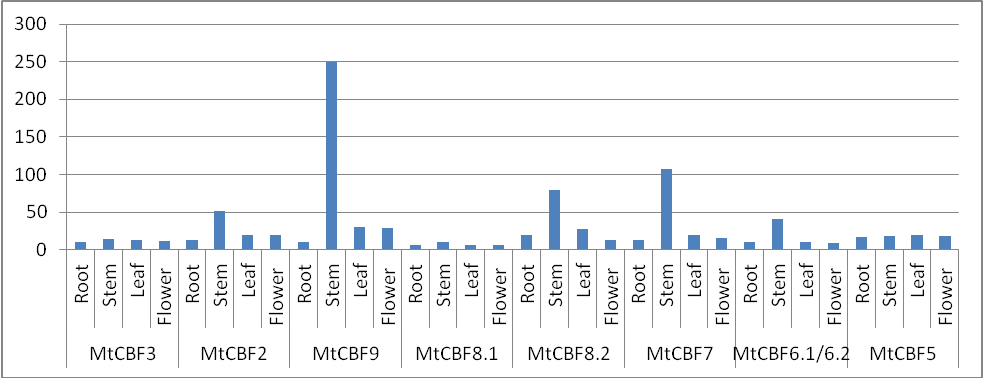


A subset of the *CBF/DREB1* genes that were identified in this study (viz. *MtCBF2*, *MtCBF3*, *MtCBF5*, *MtCBF6*, *MtCBF7*, *MtCBF8* and *MtCBF9*) mapped to Affymetrix probesets from *M. truncatula* Gene Expression Atlas version 2 [76][S28]. The correspondences between these *CBF/DREB1* genes and probeset IDs are as follows: *MtCBF3*/Mtr.11241.1.S1_at; *MtCBF2*/Mtr.27925.1.S1_at; *MtCBF9*/Mtr.6877.1.S1_at; *MtCBF8.1*/Mtr.12834.1.S1_at; *MtCBF8.2*/Mtr.29314.1.S1_at; *MtCBF7*/Mtr.30181.1.S1_at; *MtCBF6.1-6.2*/Mtr.39012.1.S1_at and *MtCBF5*/Mtr.13415.1.S1_at. The mean normalized transcript levels (Arbitrary Units) of the different *CBF/DREB1* genes in four unstressed organs (root, stem, leaf and flower) and over three independent biological replicates are shown.

Supplementary reference:

S28. The *Medicago truncatula* Gene Expression Atlas [http://mtgea.noble.org/v2]
